# Time-series analysis of pollen concentration effects on allergic conjunctivitis healthcare visits in Beijing, 2023–2024

**DOI:** 10.3389/fpubh.2026.1773333

**Published:** 2026-04-15

**Authors:** Aizhu Liu, Lei Cheng, Haijing Zhang, Rui Zhang, Xianshi Tang, Weixuan Sheng

**Affiliations:** 1 Department of Otolaryngology Head and Neck Surgery, Capital Medical University Affiliated Beijing Shijitan Hospital, Beijing, China; 2 Education Department of Capital Medical University Affiliated Beijing Shijitan Hospital, Beijing, China; 3 Department of Obstetrics and Gynecology, Beijing Shijitan Hospital, Capital Medical University, Beijing, China; 4 Key Laboratory of National Health Commission on Parasitic Disease Control and Prevention, Key Laboratory of Jiangsu Province on Parasite and Vector Control Technology, Jiangsu Institute of Parasitic Diseases, Wuxi, China; 5 Department of Anesthesiology, Capital Medical University Affiliated Beijing Shijitan Hospital, Beijing, China

**Keywords:** allergic conjunctivitis, environmental factors, generalized additive model, Pollen concentration, time series analysis

## Abstract

**Objective:**

To analyze the lagged effects of pollen concentration on allergic conjunctivitis (AC) healthcare visits and investigate the influence of environmental factors and pathogen infections on AC risk.

**Methods:**

Daily AC outpatient and emergency cases from Beijing Shijitan Hospital, along with concurrent meteorological and pollen concentration data from March 1 to October 31 in 2023–2024, were collected. Spearman rank correlation analysis, generalized additive models (GAM), and distributed lag non-linear models (DLNM) were employed to plot lag-response curves for specific cumulative effects and incremental cumulative effects of relative risks.

**Results:**

AC visits significantly increased during spring and autumn, closely aligning with peak pollen concentrations (April in spring, September in autumn). Spearman correlation analysis revealed a strong positive association between pollen concentration and AC visits (2023: *r* = 0.599; 2024: *r* = 0.637), while meteorological factors (including temperature, air pressure, etc.) showed weaker correlations. Lagged effect analysis demonstrated that the AC lag effect associated with pollen exposure in 2024 was significantly stronger than in 2023, displaying a strict dose-response relationship with pollen concentration levels. The peak value increase from baseline in relative risk (RR) for specific cumulative effects was 2.6 times higher (0.0018 in 2024 vs. 0.0007 in 2023), with a 1.9-fold longer lag duration (28 days vs. 15 days). For incremental cumulative effects, the time to peak doubled (day 28 in 2024 vs. day 15 in 2023), and the lag duration exceeded 50 days in 2024 (vs. 27 days in 2023). The lag-response curve exhibited biphasic peaks in 2024, contrasting with the unimodal pattern in 2023.

**Conclusion:**

This study confirms that pollen concentration is the dominant factor affecting the lagged effects of AC visits, with a clear dose-response relationship. The findings provide a scientific basis for AC prevention strategies and public health early-warning systems.

## 1 Introduction

Allergic conjunctivitis (AC) is an IgE-mediated type I hypersensitivity disorder with a global prevalence of 10%−20% (1). In China, the number of AC patients reached 295 million in 2020, with an annual growth rate of 0.3% (2). The characteristic clinical manifestations of AC include ocular itching, foreign body sensation, serous or mucoid discharge, conjunctival hyperemia, and papillary hyperplasia of the palpebral conjunctiva (3), which significantly impair patients' quality of life and may lead to irreversible visual impairment in severe cases (4). Its pathogenesis involves the binding of allergens to the conjunctiva, which triggers a Th2 cell-mediated immune response that stimulates B cells to produce IgE. Subsequently, the IgE binds to the surface of mast cells and activates the release of inflammatory mediators (5). Pollen serves as the primary outdoor allergen for AC (6). Climatic factors that enhance pollen production and distribution (e.g., temperature, humidity, wind speed) may increase population exposure intensity and disease risk (7). And air pollutants [including PM_2_._5_/PM_10_, O_3_, NO_2_, SO_2_, and VOCs (Volatile Organic Compounds)] in the outdoor environments can not only directly irritate the conjunctiva to induce non-allergic inflammation but also act as adjuvants to enhance immune responses to allergens (8, 9). Whereas indoor allergens encompass dust mites, pet dander, cockroaches, and molds (10, 11), with chemicals released from building materials, furniture, and cleaning products (e.g., formaldehyde, VOCs) either directly irritating the conjunctiva or exerting adjuvant effects (12). Additionally, pathogens causing acute respiratory infections (ARI), such as SARS-CoV-2, may also induce conjunctivitis symptoms (13). This study employs a time-series generalized additive model to analyze the lagged effects of pollen concentration on AC outpatient visits in Beijing during 2023–2024. By comparing annual variations in environmental exposure parameters (pollen concentration, meteorological factors) and ARI-related factors, we investigate their impacts on AC risks to provide evidence-based support for regional public health interventions.

## 2 Materials and methods

### 2.1 Data sources

Outpatient and emergency records were collected from Beijing Shijitan Hospital between March 1st and October 31st for the years 2023 and 2024. Patients diagnosed with allergic conjunctivitis (coded as “Allergic conjunctivitis” or “Atopic conjunctivitis” per ICD-10 criteria) were identified through the hospital's electronic system. AC diagnostic criteria reference: expert consensus on diagnosis and treatment of allergic conjunctivitis in China (2018). The medical records included visit dates and gender. Following strict inclusion and exclusion criteria, a total of 47,672 cases were enrolled. The corresponding meteorological data during the same periods were obtained from the Beijing Meteorological Bureau, encompassing: daily mean temperature (°F), Dew point (°F), Mean humidity (%), Average wind speed (mph), Mean atmospheric pressure (in Hg), Total precipitation (inches), Pollen concentration (grains/1000 mm^2^, from multiple plant species).

### 2.2 Statistical analysis

Descriptive statistical analysis of meteorological data and AC visit records was performed using Excel 2019 software. *R* software (version 4.1.2) was employed to analyze the temporal distribution of different pollen types, visualized via stacked area plots. Spearman's rank correlation analysis was conducted to examine pairwise associations between meteorological factors and AC visits, with a significance level set at 0.05. A cross-basis matrix incorporating pollen concentration, temperature, and humidity was constructed using the nlme, mgcv, dlnm, and splines packages to develop a generalized additive model. Environmental indicators were subsequently integrated into the Distributed Lag Non-linear Model (DLNM). The analysis included plotting lag-response curves for both relative risk-specific cumulative effects and incremental cumulative effects, generating predictive response graphs for exposure lag effects. In the generalized additive model (GAM), temporal smoothing degrees of freedom (df) were optimized via partial autocorrelation function (PACF) analysis (maximum lag = 30 days). The optimal df (21 for 2023 and 2024) minimized PACF residual absolute sums. Fit the data for 2023 and 2024, respectively using the values of 14, 21, 28, and 35. When the cumulative lag reaches a peak, select the smallest lag period for modeling.

As shown in Table 1, a total of 47,672 patients were collected during the pollen dispersal season, including 22,131 in 2023 and 25,542 in 2024. Males accounted for 43% (2023) and 44% (2024), with females comprising 57% and 56%, respectively. The peak pollen concentration was 4061 grains/1000 mm^2^ on March 11, 2023, and 7792 grains/1000 mm^2^ on March 23, 2024

**Table 1:** Statistical description of atmospheric factors, pollen concentration, and AC visits (2023–2024).

Factor	Unit	Minimum		Maximum		Mean		SD	
		2023	2024	2023	2024	2023	2024	2023	2024
Temperature	Fahrenheit degree, °F	41.6	32	92.3	88.9	69.51765	69.22963	12.5332	12.70553
Dew	°F	−9.9	−0.6	81.9	80	48.6756	51.41274	19.17509	18.38474
Humidity	%	8.9	12.8	99.8	96.5	54.18115	58.81639	20.23721	19.10326
Wind	miles per hour, mph	1.4	1.5	9.6	8.9	4.2699	4.06388	1.473857	1.399607
Press	Hg	26.2	26.1	30.3	30.5	29.7307	29.71204	0.319351	0.400438
Precipitation	Inches	0	0	5.29	5.02	0.185435	0.264176	0.641604	0.763053
Pollen	grains/1,000 mm^2^	0	0	4,061	7,792	244.5	375.42	463.159	752.13
Male		0	0	201	281	38.82	45.75	29.859	38.855
Female		2	0	251	337	51.51	58.5	35.903	46.13
Total number of patients		3	1	452	618	90.33	104.25	65	84.205

## 3 Results

### 3.1 Statistical description of atmospheric factors, pollen concentration, and AC visits

**Figure 1:**
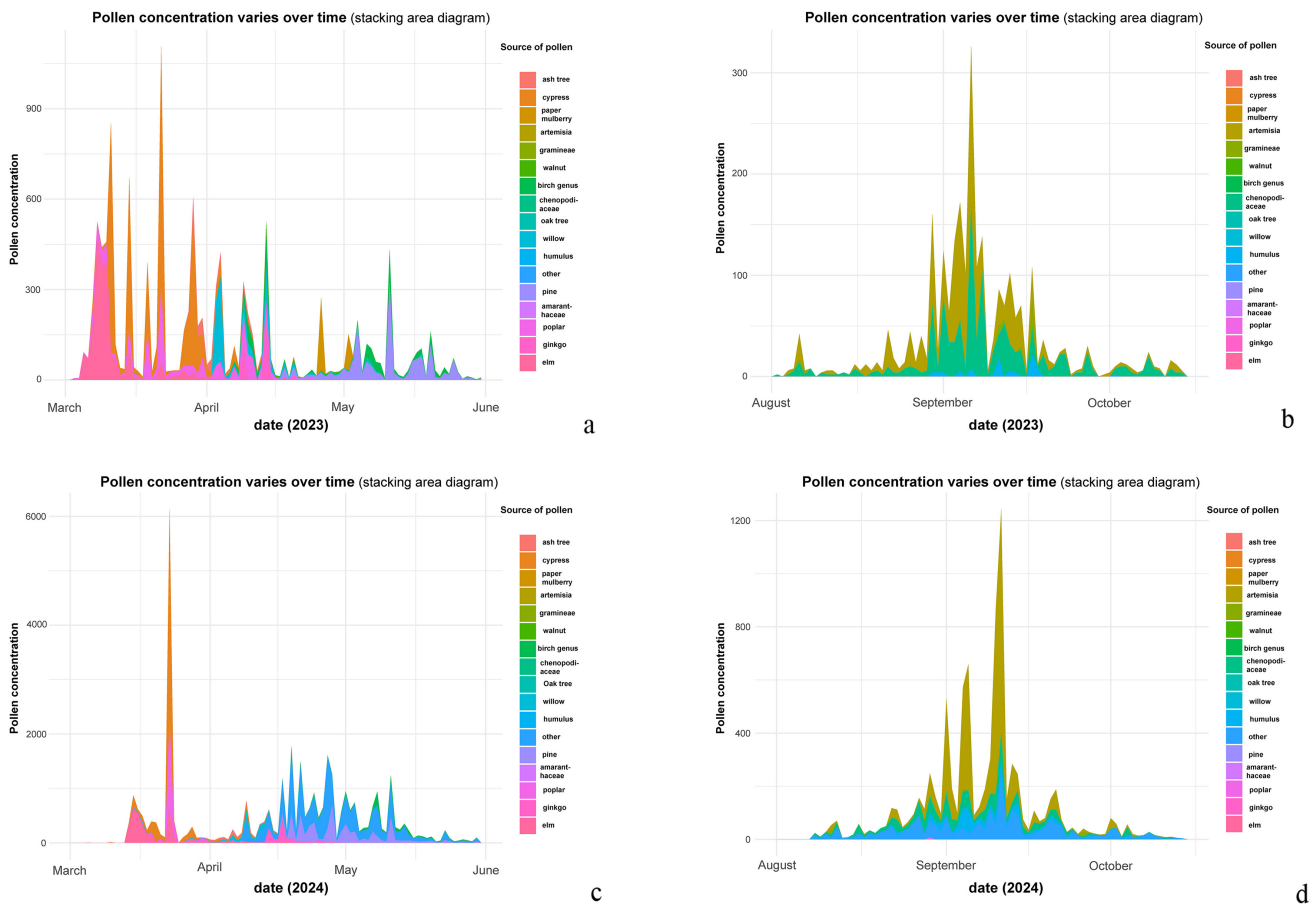
Temporal distribution of different pollen types (2023–2024). **(a)** Pollen concentration varies over time (stacking area diagram, March to June 2023). **(b)** Pollen concentration varies over time (stacking area diagram, August to October 2023). **(c)** Pollen concentration varies over time (stacking area diagram, March to June 2024). **(d)** Pollen concentration varies over time (stacking area diagram, August to October 2024).

As shown in Figure 1, distinct plant pollen types exhibited specific dispersal peaks and duration periods. Notably, variations existed between consecutive years during the same seasonal intervals. In spring (Figures 1a, c), Cupressaceae and Populus pollen dominated, whereas Artemisia species prevailed during autumn (Figures 1b, d) in both years.

**Figure 2:**
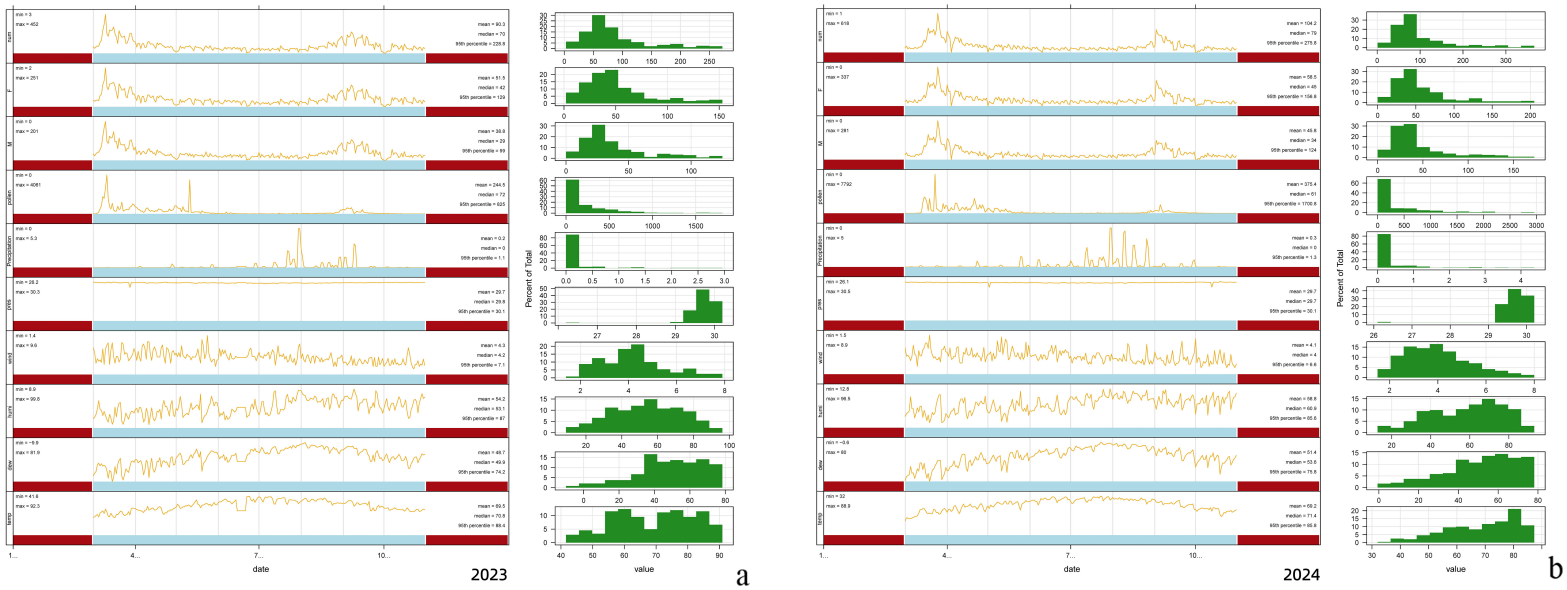
Temporal variations of meteorological parameters, pollen concentrations, and AC visits (2023–2024). **(a)** Temporal variations of meteorological parameters, pollen concentrations, and AC visits (2023). **(b)** Temporal variations of meteorological parameters, pollen concentrations, and AC visits (2024).

As shown in Figure 2a (2023) and Figure 2b (2024), AC patient visits peaked during April/May and August/September each year, demonstrating strong seasonality concentrated in spring and autumn. Pollen concentrations also exhibited pronounced seasonal trends, with the dominant spring peak in April and a secondary autumn peak in September, temporally aligned with visit surges.

### 3.2 Correlation coefficients of atmospheric factors, pollen concentrations, and AC visits

As shown in Table 2, in 2023, temperature exhibited a moderate negative correlation with patient visits (−0.372, *P* < 0.01), while dew point temperature showed a weak negative correlation (−0.249, *P* < 0.01). No statistically significant association was observed for relative humidity or average wind speed. Mean atmospheric pressure demonstrated a moderate positive correlation (0.309, *P* < 0.01), whereas precipitation revealed a weak negative correlation (−0.181, *P* < 0.01). Notably, pollen concentrations showed a moderate positive correlation (0.599, *P* < 0.01) with visit frequency.

**Table 2:** Spearman correlation coefficients between atmospheric factors, pollen concentrations, and AC visits.

Factor	Temperature		Dew		Humidity		Wind		Press		Precipitation		Pollen		Total number of patients	
	2023	2024	2023	2024	2023	2024	2023	2024	2023	2024	2023	2024	2023	2024	2023	2024
Temperature	1	1	0.783^**^	0.821^**^	0.225^**^	0.245^**^	0.049	−0.008	−0.757^**^	−0.770^**^	0.103	0.216^**^	−0.318^**^	V0.204^**^	−0.372^**^	−0.253^**^
Dew	0.783^**^	0.821^**^	1	1	0.761^**^	0.727^**^	−0.297^**^	−0.353^**^	−0.582^**^	−0.660^**^	0.328^**^	0.425^**^	−0.343^**^	−0.300^**^	−0.249^**^	−0.286^**^
Humidity	0.225^**^	0.245^**^	0.761^**^	0.727^**^	1	1	−0.563^**^	−0.631^**^	−0.142^*^	−0.210^**^	0.419^**^	0.498^**^	−0.250^**^	−0.341^**^	−0.059	−0.242^**^
Wind	0.049	−0.008	−0.297^**^	−0.353^**^	−0.563^**^	−0.631^**^	1	1	−0.162^*^	−0.042	−0.068	−0.135^*^	0.220^**^	0.234^**^	−0.048	0.123
Press	−0.757^**^	−0.770^**^	−0.582^**^	−0.660^**^	−0.142^*^	−0.210^**^	−0.162^*^	−0.042	1	1	−0.162^*^	−0.301^**^	0.166^**^	0.12	0.309^**^	0.208^**^
Precipitation	0.103	0.216^**^	0.328^**^	0.425^**^	0.419^**^	0.498^**^	−0.068	−0.135^*^	−0.162^*^	−0.301^**^	1	1	−0.130^*^	−0.159^*^	−0.181^**^	−0.145^*^
Pollen	−0.318^**^	−0.204^**^	0.343^**^	−0.300^**^	−0.250^**^	−0.341^**^	0.220^**^	0.234^**^	0.166^**^	0.12	−0.130^*^	−0.0159^*^	1	1	0.599^**^	0.637^**^
Total number of patients	−0.372^**^	−0.253^**^	−0.249^**^	−0.286^**^	−0.059	−0.242^**^	−0.048	0.123	0.309^**^	0.208^**^	−0.181^**^	−0.145^*^	0.599^**^	0.637^**^	1	1

^*^P < 0.05; ^**^P < 0.01.

In 2024, temperature showed a weak negative correlation with patient visits (−0.253, *P* < 0.01), as did dew point (−0.286, *P* < 0.01) and relative humidity (−0.242, *P* < 0.01). No statistically significant association was found for average wind speed. Mean atmospheric pressure demonstrated a weak positive correlation (0.208, *P* < 0.01), while precipitation exhibited a weak negative correlation (−0.145, *P* < 0.05). Notably, pollen concentrations displayed a strong positive correlation (0.637, *P* < 0.01) with AC visits.

**Figure 3:**
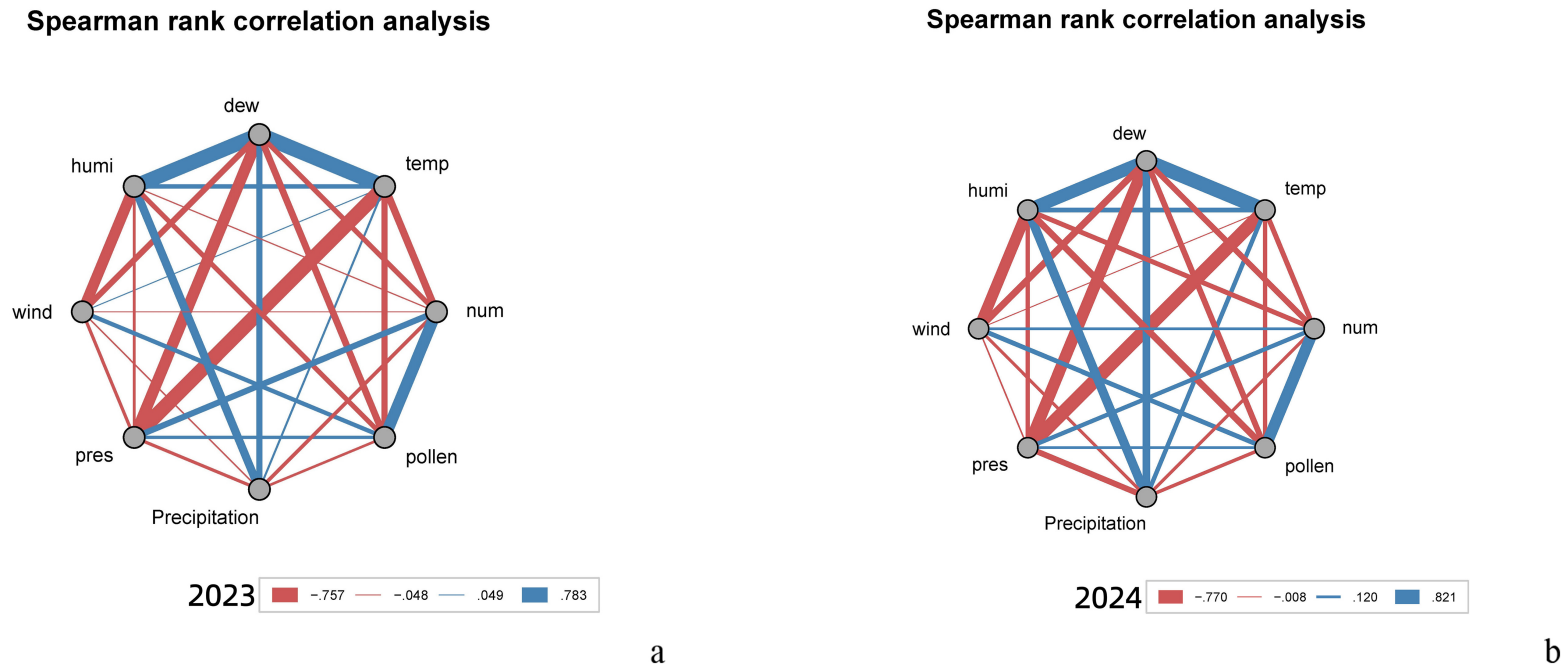
Correlation matrix. **(a)** Spearman rank correlation analysis (2023). **(b)** Spearman rank correlation analysis (2024).

Figure 3 presents a correlation matrix of all related factors, with red indicating negative correlations (peaking at −0.757 in Figure 3a and −0.770 in Figure 3b) and blue representing positive correlations (maximum values of 0.783 in Figure 3a and 0.821 in Figure 3b).

### 3.3 Lag effects

**Figure 4:**
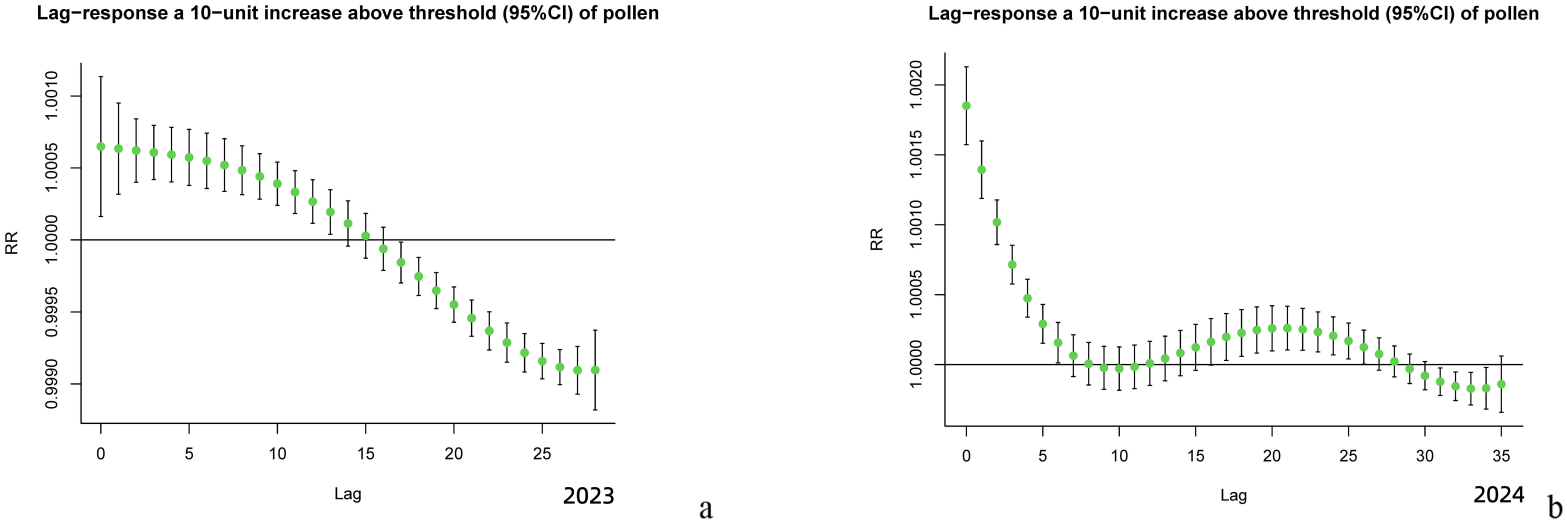
Specific Lag Effects of Pollen Exposure. **(a)** Lag-response a 10-unit increase above threshold (95%CI) of pollen (2023) . **(b)** Lag-response a 10-unit increase above threshold (95%CI) of pollen (2024).

Figure 4 shows that in 2023 (Figure 4a), each 10-unit increase in pollen concentration resulted in a specific cumulative effect that peaked at day 0, diminishing by day14, with an effect duration of 15 days and the unimodal pattern with consistent daily decline. In 2024 (Figure 4b), the same pollen concentration increment produced a peak effect on day 0, followed by an initial decline and a subsequent rise, a secondary minor peak emerging on day 21. The biphasic lag effect disappeared by day 27, resulting in a 28-day cumulative effect duration.

**Figure 5:**
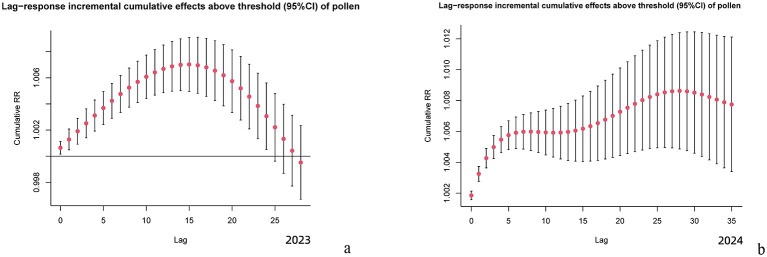
Incremental Lag Effects of Pollen Exposure. **(a)** Lag-response incremental cumulative effects above threshold (95%CI) of pollen (2023). **(b)** Lag-response incremental cumulative effects above threshold (95%CI) of pollen (2024).

Figure 5 demonstrates that in 2023 (Figure 5a), each 10-unit increase in pollen concentration lead to a peak incremental cumulative effect on day 14, diminishing by day 27, with a total effect duration of 28 days, exhibiting a unimodal trend. In 2024 (Figure 5b), the same exposure showed a peak effect at day 28, with persistent lag effects remaining detectable beyond day 35 and a total effect duration exceeding 50 days, displaying a multimodal pattern.

**Figure 6:**
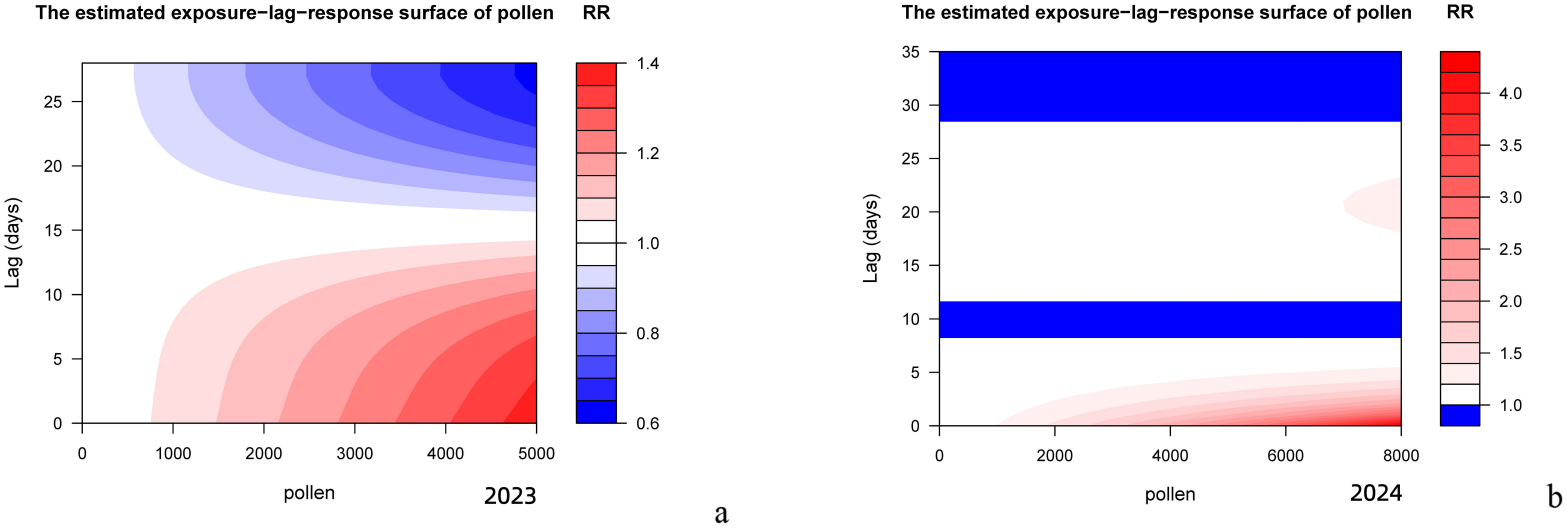
Pollen Concentration Contour Map. **(a)** The estimated exposure-lag-response surface of pollen (2023). **(b)** The estimated exposure?lag?response surface of pollen (2024).

Figure 6a (2023) and Figure 6b (2024) demonstrate that higher pollen concentrations were associated with stronger lag effects and prolonged effect durations.

## 4 Discussion

Using a distributed lag non-linear model, this study revealed significant differences in the lag effects of AC risk per 10-unit increase in pollen concentration between 2023 and 2024. For specific cumulative lag effects, the peak relative risk value increase from baseline (1.0000) reached 0.0018 in 2024, 2.6 times higher than the 0.0007 observed in 2023. Concurrently, the duration of elevated risk above baseline extended from 15 days in 2023 to 28 days in 2024, with a 1.9-fold increase. Regarding incremental cumulative lag effects, the peak relative risk value rose to 1.009 in 2024 compared to 1.007 in 2023, with the time to peak doubling to 28 days (vs. 15 days in 2023). Furthermore, the duration of AC risk elevation exceeded 50 days in 2024, nearly twice the 27-day duration in 2023. And the lag curve morphology also showed marked differences: while 2023 exhibited a unimodal curve with monotonic decline after peak exposure, 2024 demonstrated multiphasic characteristics featuring a secondary peak around day 20 on the specific lag effect curve in addition to the initial peak, suggesting that high pollen concentrations might trigger secondary immune responses. These annual differences in lag effects correlated strongly with substantial variations in pollen concentrations between the 2 years. Although pollen species composition remained similar between 2023 and 2024, dominated by arboreal pollens (cypress, elm, poplar, willow, pine, ginkgo, and birch) in spring (March–May) and herbaceous pollens (Artemisia and Chenopodiaceae) in autumn (August–October), the peak pollen concentration surged by 92% from 4,061 grains/mm^2^ in 2023 to 7,792 grains/mm^2^ in 2024, with annual mean concentrations increasing 1.5-fold from 244.54 ± 63.16 grains/mm^2^ (2023) to 375.427 ± 52.13 grains/mm^2^ (2024). This concentration-lag effect relationship was further validated by 2018–2019 historical data: when annual pollen concentrations were comparable (2018: 109.741 ± 47.73 grains/mm^2^; 2019: 87.831 ± 05.60 grains/mm^2^), the lag durations showed similar lengths (specific lag: 10 vs. 9 days; incremental lag: 21 vs. 22 days), both presenting unimodal curves (14). These findings confirm a strict dose-response relationship between pollen concentration levels and both the intensity and duration of lag effects. Notably, against the background of no significant differences in meteorological parameters' correlation with AC visits and no reported dramatic fluctuations in outdoor air pollutants or indoor allergens like mites in Beijing between 2023–2024, these results strongly suggest pollen concentration as the dominant factor influencing AC risk lag effects under the conditions set forth in this study.

In addition to differences in annual pollen concentrations, significant variations were observed in the epidemiological characteristics and pathogen composition of ARI between 2023 and 2024. Following the discontinuation of China's zero-COVID policy in late 2022, SARS-CoV-2 infection rates peaked rapidly across China afterwards, including Beijing. A multicenter cross-sectional study conducted in Jiangsu Province, involving 1,065 children under 12 years old from 7 hospitals across 6 regions, revealed that over 90% of sampled children had been infected with SARS-CoV-2 during February 10 to March 10, 2023 (15). Another retrospective single-center study on respiratory pathogens in Beijing reported a SARS-CoV-2 infection rate of 35.0% in May 2023, which remained significantly higher than the 2024 sampling results (23.7% in March and 20.9% in August) (16). Furthermore, as SARS-CoV-2 continued to evolve, the predominant variants in 2024 (XBB and BA.2.86) demonstrated lower pathogenicity compared to the early 2023 dominant variant (BA.5.2), with significantly reduced incidence of severe cases and predominantly mild symptoms among infected patients (17). Beyond SARS-CoV-2, during the 2023 autumn pollen season (September-October), the detection rates of human rhinovirus (HRV) and Mycoplasma pneumoniae (MP) in Beijing ranged between 20%−50%, before declining to 5%−15% from November 2023 to February 2024; Adenovirus (ADV) and respiratory syncytial virus (RSV) maintained stable detection rates of 10%−20% throughout both years (18). Notably, seasonal influenza viruses (influenza A and B), which typically peak between October and February, showed no temporal overlap with the pollen season (19).

Multiple respiratory pathogens can cause ocular infections, with viral conjunctivitis being the most common manifestation (20). The ocular surface - particularly the corneal and conjunctival epithelium - serves as a mucosal gateway for viral invasion (21–23). Beyond SARS-CoV-2, pathogens including mumps virus (24), respiratory syncytial virus (25), and influenza A H7 subtype (26) have been documented to cause human conjunctivitis. Among COVID-19 patients, conjunctivitis incidence ranges from 0.8% to 4.8% (27, 28), with rare cases reporting conjunctivitis as the initial presenting symptom (29). However, our study found that even during the 2023 peak of SARS-CoV-2 and other respiratory infections, these pathogens showed no significant exacerbating effect on AC lagged responses, with their impact substantially weaker than that of pollen concentration variations. This phenomenon may be attributed to the dynamic protective mechanisms of the tear film (30). Besides, the continuous tear secretion helps flush pathogens into the nasolacrimal system (31, 32), evidenced by the minimal SARS-CoV-2 RNA detection rates (0%−5.2%) in conjunctival swabs or tears from COVID-19 patients (33, 34). Furthermore, the limited susceptibility of conjunctival epithelial cells plays a crucial role: single-cell sequencing has confirmed significantly lower expression of SARS-CoV-2 binding receptors (ACE2) and the protease TMPRSS2 on ocular surfaces compared to respiratory tissues (35, 36), consistent with clinical observations of rapid conjunctivitis resolution following topical antiviral treatment in COVID-19 cases (20).

## 5 Conclusion

Through the construction of generalized additive models, pollen concentration was identified as the dominant factor influencing the lagged effects of AC healthcare visits under the conditions set forth in this study, and ARI do not serve as key modulators of AC lagged response variations. These findings provide crucial scientific evidence for developing pollen-related AC prevention strategies and dynamically adjusting public health early-warning policies. The limitations of this study include: relying solely on single-center data without capturing regional population heterogeneity; failing to quantify individual infection status to assess its impact on AC risk variation; not thoroughly differentiating the independent effects of various pathogens or identifying specific pathogen-pollen exposure synergies; and possibly with unaccounted confounding factors in the statistical models that could potentially influence the results. Future research would incorporate multicenter clinical data and real-time infection surveillance metrics, refine ARI pathogen classification to clarify distinct pathogens' independent effects on AC lagged responses, conduct cohort studies to collect detailed individual-level data including pollen exposure levels, allergy history and protective behaviors for analyzing personalized AC risk profiles, and further optimize statistical models by including potential confounders such as population immunity levels, urban green space distribution, pollen-air pollutant interaction and indoor environmental quality.

## Data Availability

The raw data supporting the conclusions of this article will be made available by the authors, without undue reservation.
